# Metastatic cerebellar tumor of papillary thyroid carcinoma mimicking cerebellar hemangioblastoma

**DOI:** 10.1186/s40064-016-2551-4

**Published:** 2016-06-29

**Authors:** Makoto Ideguchi, Takafumi Nishizaki, Norio Ikeda, Shigeki Nakano, Tomomi Okamura, Natsumi Fujii, Tokuhiro Kimura, Eiji Ikeda

**Affiliations:** Department of Neurosurgery, Ube-kohsan Central Hospital, 750 Nishikiwa, Ube, Yamaguchi 755-0151 Japan; Department of Neurosurgery, Yamaguchi University Graduate School of Medicine, Ube, Japan; Department of Pathology, Yamaguchi University Graduate School of Medicine, Ube, Japan

**Keywords:** Metastatic cerebellar tumor, Papillary thyroid carcinoma, Cerebellar hemangioblastoma, Challenging diagnosis, Solid tumor

## Abstract

**Introduction:**

Well-differentiated papillary thyroid carcinoma generally (PTC) have a favorable prognosis. This metastasis is rare in the central nervous system. Brain metastasis has a relatively poor prognosis. We present a rare case of cerebellar metastasis, one that mimics a solid type cerebellar hemangioblastoma and because of which it was very hard to reach accurate preoperative diagnosis. Accurate diagnosis was challenging because of the similar imaging and histopathological findings for these two tumors.

**Case description:**

A brain lesion was detected by routine medical checkup of the brain with MRI in a 49-year-old woman 2 years after thyroidectomy for well-differentiated PTC. Gadolinium-enhanced MRI showed a homogeneous prominently enhanced lesion with surrounding enhanced dilated vessels in the left cerebellar hemisphere. Digital subtraction angiography showed a strongly stained lesion fed by the peripheral branch of the left posterior inferior cerebellar artery with drainage into the inferior vermian vein, revealing arteriovenous shunting. The most like likely preoperative diagnosis was felt to be that of a solid cerebellar hemangioblastoma. Gross total resection of the tumor was achieved by bilateral suboccipital craniotomy, and intraoperative pathological analysis suggested hemangioblastoma. Histopathological findings showed proliferation of vacuolated sheeted tumor cells with clear and eosinophilic cytoplasm and numerous thin-walled microvessels, consistent with hemangioblastoma. However, the final diagnosis was brain metastasis of the follicular variant of PTC due to a partial thyroid follicle-like pattern including eosinophilic fluid pathologically and positive TTF-1 immunostaining.

**Discussion and evaluation:**

Since presented rare case of cerebellar metastasis of PTC was very similar to solid type cerebellar hemangioblastoma on imaging and histopathological findings, accurate diagnosis was challenging. Moreover, it is extremely rare for a cerebellar metastasis to occur as an initial distant metastasis of PTC, and hemangioblastoma is the most common primary cerebellar neoplasm in adults. This epidemiological data was also one of the reason of difficulty to reach preoperative accurate diagnosis.

**Conclusions:**

To the best of our knowledge, there are no other reports of challenging diagnosis case of these two tumors in the literature. Brain metastasis of a well-differentiated PTC could be a relatively poor prognostic factor, and accurate diagnosis and suitable surgical therapy or radiotherapy are needed.

## Background

Papillary thyroid carcinoma (PTC) is the most common thyroid carcinoma, representing approximately 80 % of newly diagnosed thyroid carcinomas (Hjiyiannakis et al. 1996). PTCs are characterized by a slowly progressive course and a 10-year survival rate of 80–95 % (Schlumberger 1998). Distant metastases are seen in a minority of patients, at rates of 4–15 % (Hoie et al. 1988; Casara et al. 1993; Shaha et al. 1997; Clark et al. 2005; Aggarwal et al. 2007), and are most common in the lungs, followed by bone (Mazzaferri and Massoll 2002). Brain metastases are rare, occurring in roughly 0.15–1.3 % of thyroid carcinomas (Parker et al. 1986). Here, we present a case with brain metastasis of PTC that mimicked cerebellar hemangioblastoma radiographically and pathologically. This case illustrates the challenge of differential diagnosis of cerebellar hemangioblastoma and a metastatic cerebellar tumor of PTC.

## Case description

**Fig. 1:**
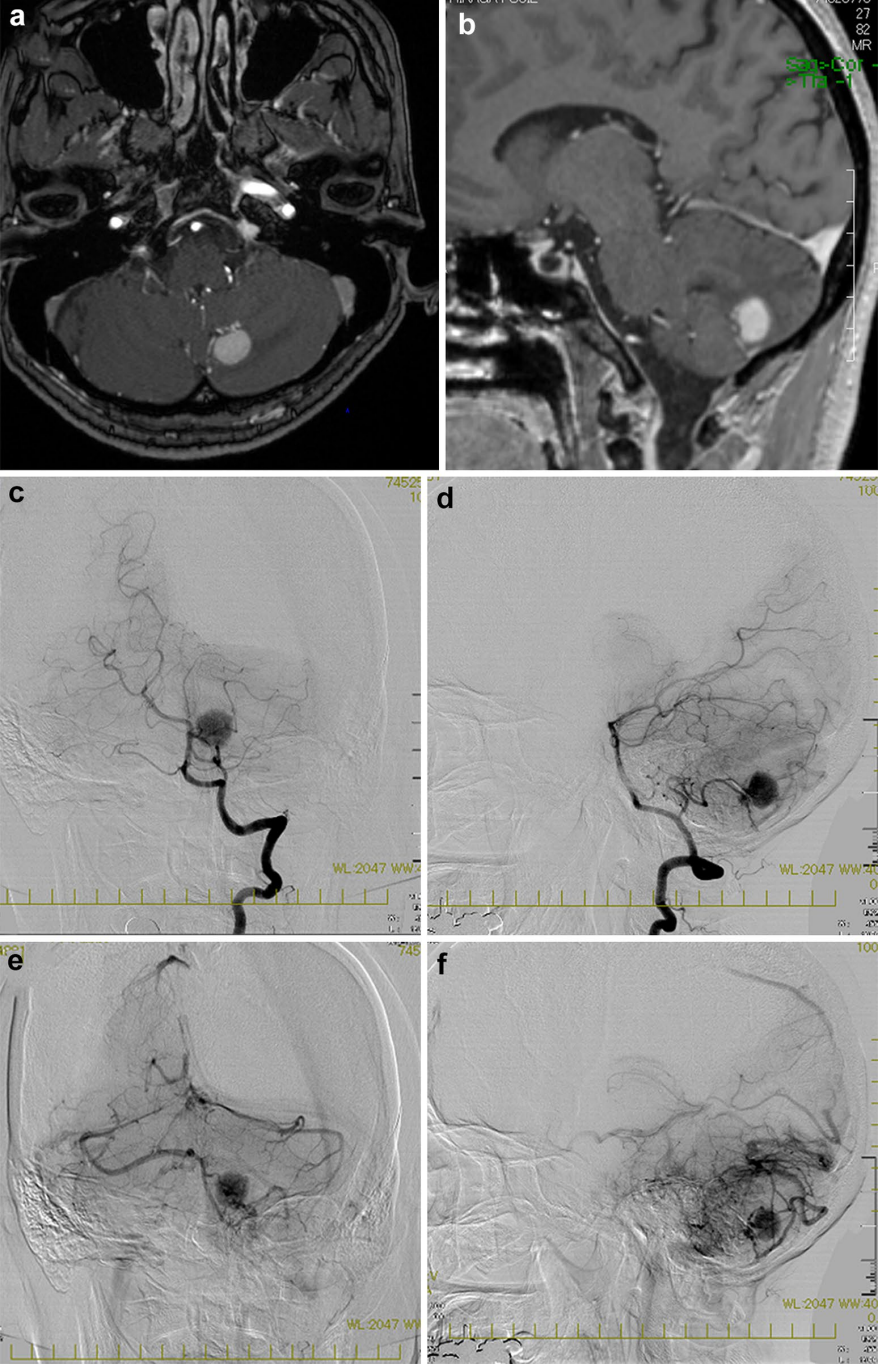
Preoperative magnetic resonance imaging and digital subtraction angiography. Preoperative axial (**a**) and sagittal (**b**) views in gadolinium-enhanced T1-weighted MRI, showing a prominent enhanced mass lesion and enhanced vessel-like structures in surrounding parenchyma in the left cerebellar hemisphere. Anteroposterior (**c**) and lateral (**d**) views in angiography, showing strong tumor staining with a feeding artery from the left posterior inferior cerebellar artery, and a draining vein into the left inferior vermian vein, indicating arteriovenous shunt [anteroposterior view (**e**) and lateral view (**f**)]

A 49-year-old woman visited our institute for an incidentally detected brain tumor during the routine medical checkup of the brain with MRI. She had a history of hysterectomy for uterine myoma 6 years ago and total thyroidectomy for thyroid carcinoma that was diagnosed as well-differentiated PTC 2 years ago. TNM-stage was Stage III (T3N0M0) when thyroidectomy. Radioiodine treatment was not performed because of no evidence of invasion to surrounding tissue, lymph node metastasis and distal metastasis. She also had a familial history of thyroid carcinoma, since her mother had suffered from the same thyroid tumor. Physical and neurological examinations were all normal with no deficit. All serum tumor markers were within the normal range. Computed tomography showed that the tumor was a homogeneously enhanced mass lesion in the left cerebellum. Gadolinium-enhanced magnetic resonance imaging (MRI) showed a homogeneous strongly enhanced hypervascular lesion with surrounding enhanced vessels located in the left cerebellar hemisphere (Fig. 1a, b). Digital subtraction angiography showed a strongly stained lesion fed by the peripheral branch (tonsillohemispheric segment) of the left posterior inferior cerebellar artery (PICA) and draining into the inferior vermian vein, revealing arteriovenous shunting (Fig. 1c–f). Preoperative thyroglobulin (Tg) was 9.5 ng/ml (normal range: 0–50), and anti-thyroglobulin antibody (Anti-Tg antibody) measured by FEIA method was under 6.0 IU/ml (normal range: under 13.6). A pre-diagnosis of solid type cerebellar hemangioblastoma was made based on these radiographic findings and the epidemiology that it is the most common primary cerebellar neoplasm in adults (Committee of Brain Tumor Registry of Japan 2014).

**Fig. 2:**
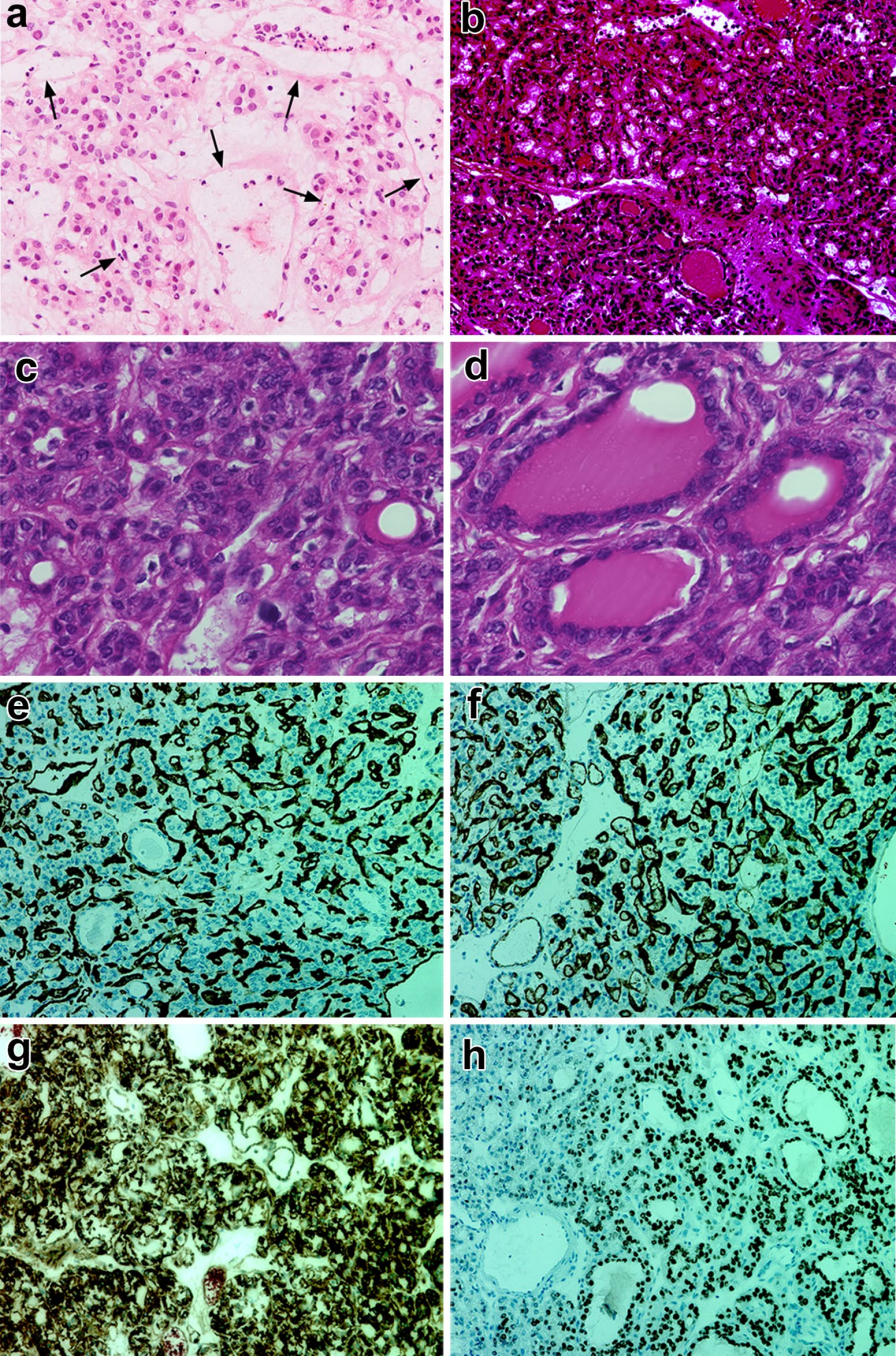
Photomicrographs showing histological and immunohistochemical findings in tumor tissue. **a** Intraoperative frozen section of the tumor tissue. Polygonal cells proliferated in nests and cords. Many blood vessels (*arrows*) were found between the nests and cords. Hematoxylin and eosin staining in the formalin-fixed paraffin-embedded tumor tissue showed numerous thin-walled microvessels (**b**) and proliferation of vacuolated atypical sheeted tumor cells with clear and eosinophilic cytoplasm (**c**). **d** A small part of the tumor had a thyroid follicle-like pattern including eosinophilic liquid. CD31 (**e**) and CD34 (**f**) staining were positive. **g** The tumor was diffusely positive for vimentin. **h** Thyroid transcription factor-1 (TTF-1)-positive cells were present in part of the tumor. **a**, **b**, **e**–**h** ×200; **c**, **d** ×600

We performed tumor resection by bilateral suboccipital approach. By opening the arachnoid membrane between the vermis and left biventral lobule, a long perspective from the proximal to distal portion of the PICA across the predicted location of the tumor bulk was exposed. The tumor position was identified using a navigation system, and the exposed well-margined tumor bulk was totally resected after dissecting the feeding artery from the PICA peripheral branch and the draining vein into the left inferior vermian vein. An intraoperative pathological examination was performed, and the frozen section of the tumor tissue showed that polygonal cells proliferated in nests and cords with many blood vessels (Fig. 2a). The findings appeared compatible with hemangioblastoma. Postoperatively, the patient had no physical or neurological symptoms.

Histological examination of the formalin-fixed paraffin-embedded tumor tissue revealed proliferation of vacuolated sheeted tumor cells with clear and eosinophilic cytoplasm and numerous thin-walled vessels (Fig. 2b, c). A thyroid follicle-like pattern including eosinophilic liquid was partially present (Fig. 2d). Irregular nuclear contours and nuclear grooves were focally observed. Immunohistochemical findings were positive for CD31 (dilution 1:40; Dako, Japan), CD34 (dilution 1:50; Nichirei, Tokyo, Japan), vimentin (dilution 1:50; Dako) and thyroid transcription factor-1 (TTF-1) (dilution 1:200; Dako) (Fig. 2e–h), and negative for GFAP (data not shown). The final diagnosis was PTC.

**Fig. 3:**
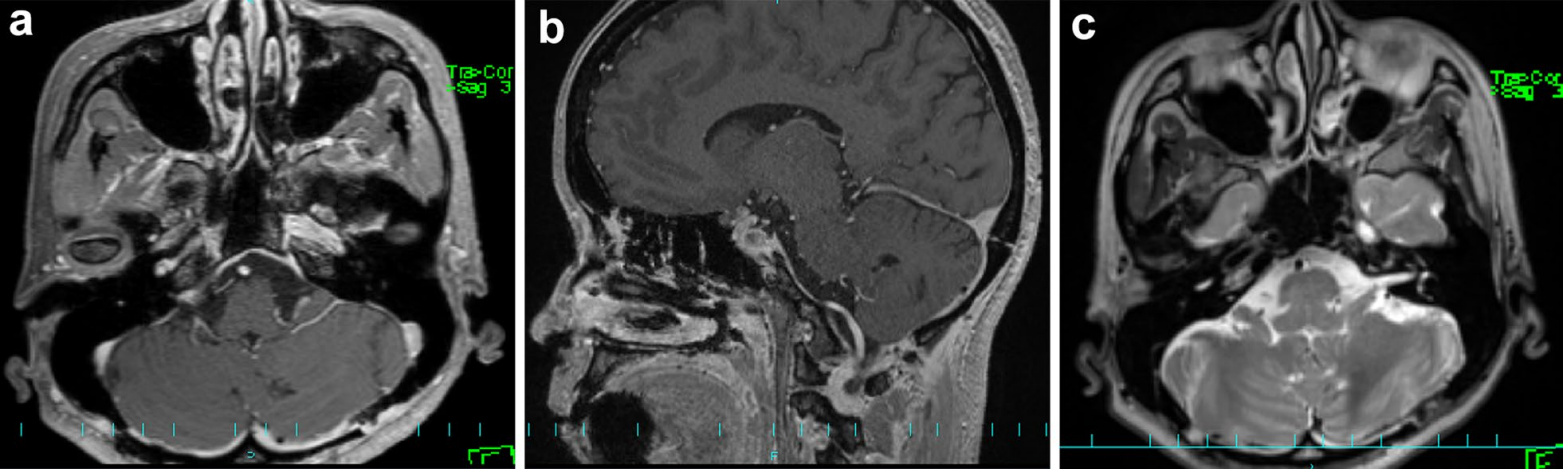
Postoperative magnetic resonance imaging. Postoperative axial (**a**) and sagittal (**b**) views of gadolinium-enhanced T1-weighted and **c** T2-weighted MRI, showing total resection of the tumor and no ischemic and contusional complications

Postoperative MRI showed total resection of the tumor bulk and no ischemic complications (Fig. 3). Therefore, postoperative radiotherapy and adjuvant therapy were not administered. At 2 years postoperatively, the patient has no symptoms and no recurrence on follow-up MRI. Tg and anti-Tg antibody 2 months and 2 years after operation were under 0.1 ng/ml and under 6.0 IU/ml, 0.11 ng/ml and under 6.0 IU/ml, respectively, which all were normal range.

## Discussion and evaluation

Papillary thyroid carcinoma commonly metastasizes to regional lymph nodes, lungs, and bones, whereas the brain is an uncommon site of metastasis that is found in only 0.15–1.3 % of cases (Parker et al. 1986; Tanaka et al. 2013). Moreover, it is extremely rare for a cerebellar metastasis to occur as an initial distant metastasis of PTC, with only 16 such cases published to date (Tunio et al. 2015). A brain metastasis of thyroid cancer could be a negative prognostic factor and the median survival in such cases were reported as 4.7 months (including anaplastic type 11 cases) (n = 47) (Chiu et al. 1997), 9.4 months (n = 9) (Misaki et al. 2000), 17.4 months (n = 16) (McWilliams et al. 2003), for a maximum of 29.2 months (n = 9) (Kim et al. 2009). Chiu et al. (1997) reported that the overall median survival of patients with brain metastases from well-differentiated thyroid cancers was 12.4 months. Patients with PTC with metastases to other organs also have reduced survival after diagnosis of a brain metastasis (Biswal et al. 1994; Hjiyiannakis et al. 1996; Salvati et al. 2001; McWilliams et al. 2003; Kim et al. 2009; Tsuda et al. 2013).

There are no characteristics of MRI or other radiographic abnormalities that distinguish metastatic lesions of thyroid carcinoma from other brain tumors (McWilliams et al. 2003; Tunio et al. 2015). There are only three cases in which findings in digital subtraction angiography have been described (Cha et al. 2000; Hashiba et al. 2006; Umehara et al. 2015). In each of these, relatively prominent-tumor staining with an apparent feeding artery was observed, and the consistency of these findings with those in our case suggest that a metastatic PTC lesion has a tendency to develop into a vascular-rich tumor bulk. There have been five reports of cases exhibiting cerebellar hemorrhage secondary to brain metastasis of PTC (Mazzaferri et al. 1977; Isoda et al. 1997; Pacak et al. 1998; Lecumberri et al. 2010; Tanaka et al. 2013). McWilliams et al. (2003) reported that 10 of 16 cases were very or extremely vascular at the time of surgery or had imaging evidence of hemorrhage. This hemorrhagic feature is consistent with the angiographic findings, and indicates that brain metastasis of PTC is a relatively hypervascular tumor.

Hemangioblastoma typically occurs in the young adult and the most common posterior fossa-primary tumors, 1–2.5 % of all intracranial tumors, approximately 10 % of all posterior fossa tumors (Ho et al. 1992; Farrukh 1996) and 29 % of all cerebellar tumors (Committee of Brain Tumor Registry of Japan 2014). Intracranial hemangioblastomas can manifest as four morphologic patterns based on the macroscopic pathology. Type 1 (5 % of posterior fossa hemangioblastoma) is a simple cyst without a macroscopic nodule. Type 2 is a cyst with a mural nodule (60 %). Type 3 is a solid tumors (26 %), and type 4 is a solid tumors with small internal cysts (9 %), are also seen in the cerebellum (Lee et al. 1989; Ho et al. 1992; Richard et al. 1998). In gadolinium-enhanced MRI, the nodule has prominent enhancement (Ho et al. 1992). Since MRI findings for solid hemangioblastomas are similar to those in the present case, it was hard to make a definite diagnosis based on imaging (Hwang et al. 2015). Angiographical findings for hemangioblastoma often include enlarged feeding arteries and dilated draining veins with dense tumor staining (Ho et al. 1992; Hwang et al. 2015). Angiography in the present case showed a hypervascular mass stain with a feeding artery, a draining vein and an arteriovenous shunt that were also very similar to the common findings in hemangioblastoma, and distinguishing between these tumors was difficult. Pathologically, papillary carcinomas usually share certain features. The neoplastic papillae contain a central core of fibrovascular tissue lined by one or occasionally several layers of cells with crowded oval nuclei (LiVolsi 2011). In the present case, the papillary growth feature was not particularly apparent, but vacuolated cells with clear to eosinophilic cytoplasm grew as sheet-like structures accompanied by dense proliferation of small vessels. These features are also similar to the characteristics of hemangioblastoma. However, a small fraction of the tumor had a thyroid follicular-like pattern with hypereosinophilic colloids, which is characteristic of PTC. Immunohistochemically, papillary carcinoma is usually positive for TTF1, HBME-1, and CK19, and partially positive for vimentin, CD31 and CD34 (Tuffaha 2008). The density of CD31 and CD34 staining is associated with microvessel intensity (Jiang et al. 2014). The present case had CD31- and CD34-positive dense microvessels in the whole tumor, which was an additional reason for the difficulty of accurate diagnosis. TTF-1 was the most diagnostically positive marker in this case.

Survival is generally good in patients with differentiated PTC (Chiu et al. 1997). However, distatant metastases and local tumor infiltration can be fatal, as supported by the high mortality associated with brain metastasis of PTC (McConahey et al. 1986).

There is no clearly defined protocol for management of intracranial metastases of thyroid cancer, but surgery is generally considered to be the best choice for prolonged survival and regression of neurological symptoms (Aguiar et al. 2001; Blankenship et al. 2005; Pazaitou-Panayiotou et al. 2005; Al-Dhahri et al. 2009; Kim et al. 2009; Bernad et al. 2010). Chiu et al. (1997) reported a significant increase in median survival (16.7 months) for those patients who were performed surgical resection of their metastases, compared with 3.4 months for those who did not. Moreover, the data of McWilliams et al. showed supported that assertion, with survivals of 18.7, 25.5, and 2.7 months, respectively, reported for those patients who underwent gross total resection, partial resection, and no surgery (McWilliams et al. 2003). It is possible that radiosurgery would be generally candidate-therapy for a single, small-sized metastasis as previously described (Muacevic et al. 2008). However, since our preoperative diagnosis was hemangioblastoma, we selected surgical resection with meaning of acquiring a accurate diagnosis. In previous studies, patients undergoing stereotactic radiosurgery had overall median survival of 33–37.4 months, with gross total resection being an attractive option for local control of a solitary lesion or a few lesions (Biswal et al. 1994; Salvati et al. 2001; Al-Dhahri et al. 2009). Previously published reports have noted an apparent benefit of radioactive iodine treatment when uptake scans in the brain are positive (Hjiyiannakis et al. 1996). There is an excellent study that reports the survival of a 15-year old girl with diffuse and cerebral metastasis after she was given an additional age-adapted high-dose radioactive iodine treatment (Vrachimis et al. 2013). However, it should be paid enough attention to worsening of tumor growth during thyroid withdrawal and to aggravating of cerebral edema. Datz et al. recommended prophylactic glycerol for acute cerebral edema, rather than corticosteroids, because of the possibility of decreased iodine uptake from corticosteroids during treatment of intracranial metastases from thyroid carcinoma (Datz 1986). On the other hand, McWilliams et al. (2003) found no evidence to support use of chemotherapy in patients with brain metastasis of thyroid carcinoma. In the present case, additional therapy was not given because gross total resection was achieved, and there has been no recurrence radiologically and symptomatically in 2 years of postoperative follow up.

## Conclusions

We presented a rare case of cerebellar metastasis of PTC in which accurate diagnosis was challenging due to preoperative imaging and histopathological findings that were similar to those for solid type cerebellar hemangioblastoma. Cases with metastasis of a well-differentiated PTC generally have a favorable prognosis. However, since brain metastasis of a well-differentiated papillary thyroid carcinoma could be a relatively poor prognostic factor, accurate diagnosis and suitable surgical therapy or radiotherapy are needed. Characteristic imaging findings are not apparent, and thus the clinical course and history of treatment for thyroid carcinoma are keys for correct diagnosis. A hemorrhagic tendency and a hypervascular mass lesion might also be characteristic features of brain metastasis of PTC.

## Abbreviations

PTC: papillary thyroid carcinoma
MRI: magnetic resonance imaging
PICA: posterior inferior cerebellar artery

## References

[CR1] Aggarwal V, Bhargav PR, Mishra A, Agarwal G (2007) Clinico-pathological characteristics and long-term outcome in patients with distant metastases from differentiated thyroid carcinoma. World J Surg 31:246–247; author reply 247–248 10.1007/s00268-006-0546-y 17180558

[CR2] Aguiar PH, Agner C, Tavares FR, Yamaguchi N (2001). Unusual brain metastases from papillary thyroid carcinoma: case report. Neurosurgery.

[CR3] Al-Dhahri SF, Al-Amro AS, Al-Shakwer W, Terkawi AS (2009). Cerebellar mass as a primary presentation of papillary thyroid carcinoma: case report and literature review. Head Neck Oncol.

[CR4] Bernad DM, Sperduto PW, Souhami L, Jensen AW, Roberge D (2010). Stereotactic radiosurgery in the management of brain metastases from primary thyroid cancers. J Neurooncol.

[CR5] Biswal BM, Bal CS, Sandhu MS, Padhy AK, Rath GK (1994). Management of intracranial metastases of differentiated carcinoma of thyroid. J Neurooncol.

[CR6] Blankenship DR, Chin E, Terris DJ (2005). Contemporary management of thyroid cancer. Am J Otolaryngol.

[CR7] Casara D, Rubello D, Saladini G, Masarotto G, Favero A, Girelli ME, Busnardo B (1993). Different features of pulmonary metastases in differentiated thyroid cancer: natural history and multivariate statistical analysis of prognostic variables. J Nucl Med.

[CR8] Cha ST, Jarrahy R, Mathiesen RA, Suh R, Shahinian HK (2000). Cerebellopontine angle metastasis from papillary carcinoma of the thyroid: case report and literature review. Surg Neurol.

[CR9] Chiu AC, Delpassand ES, Sherman SI (1997). Prognosis and treatment of brain metastases in thyroid carcinoma. J Clin Endocrinol Metab.

[CR10] Clark JR, Lai P, Hall F, Borglund A, Eski S, Freeman JL (2005). Variables predicting distant metastases in thyroid cancer. Laryngoscope.

[CR11] Committee of Brain Tumor Registry of Japan (2014). Report of Brain Tumor Registry of Japan (2001–2004). Neurol Med Chir (Tokyo).

[CR12] Datz FL (1986). Cerebral edema following iodine-131 therapy for thyroid carcinoma metastatic to the brain. J Nucl Med.

[CR13] Farrukh HM (1996). Cerebellar hemangioblastoma presenting as secondary erythrocytosis and aspiration pneumonia. West J Med.

[CR14] Hashiba T, Maruno M, Fujimoto Y, Suzuki T, Wada K, Isaka T, Izumoto S, Yoshimine T (2006). Skull metastasis from papillary thyroid carcinoma accompanied by neurofibromatosis type 1 and pheochromocytoma: report of a case. Brain Tumor Pathol.

[CR15] Hjiyiannakis P, Jefferies S, Harmer CL (1996). Brain metastases in patients with differentiated thyroid carcinoma. Clin Oncol (R Coll Radiol).

[CR16] Ho VB, Smirniotopoulos JG, Murphy FM, Rushing EJ (1992). Radiologic–pathologic correlation: hemangioblastoma. AJNR Am J Neuroradiol.

[CR17] Hoie J, Stenwig AE, Kullmann G, Lindegaard M (1988). Distant metastases in papillary thyroid cancer. A review of 91 patients. Cancer.

[CR18] Hwang KJ, Song SJ, Park KC, Yoon SS, Ahn TB (2015). Solid cerebellar hemangioblastoma with peritumoral edema: 5-years follow up. Investig Magn Reson Imaging.

[CR19] Isoda H, Takahashi M, Arai T, Ramsey RG, Yokoyama T, Mochizuki T, Yamamoto I, Kaneko M (1997). Multiple haemorrhagic brain metastases from papillary thyroid cancer. Neuroradiology.

[CR20] Jiang J, Shang X, Zhang H, Ma W, Xu Y, Zhou Q, Gao Y, Yu S, Qi Y (2014). Correlation between maximum intensity and microvessel density for differentiation of malignant from benign thyroid nodules on contrast-enhanced sonography. J Ultrasound Med.

[CR21] Kim IY, Kondziolka D, Niranjan A, Flickinger JC, Lunsford LD (2009). Gamma knife radiosurgery for metastatic brain tumors from thyroid cancer. J Neurooncol.

[CR22] Lecumberri B, Alvarez-Escola C, Martin-Vaquero P, Nistal M, Martin V, Riesco-Eizaguirre G, Sosa G, Pallardo LF (2010). Solitary hemorrhagic cerebellar metastasis from occult papillary thyroid microcarcinoma. Thyroid.

[CR23] Lee SR, Sanches J, Mark AS, Dillon WP, Norman D, Newton TH (1989). Posterior fossa hemangioblastomas: MR imaging. Radiology.

[CR24] LiVolsi VA (2011). Papillary thyroid carcinoma: an update. Mod Pathol.

[CR25] Mazzaferri EL, Massoll N (2002). Management of papillary and follicular (differentiated) thyroid cancer: new paradigms using recombinant human thyrotropin. Endocr Relat Cancer.

[CR26] Mazzaferri EL, Young RL, Oertel JE, Kemmerer WT, Page CP (1977). Papillary thyroid carcinoma: the impact of therapy in 576 patients. Medicine (Baltim).

[CR27] McConahey WM, Hay ID, Woolner LB, van Heerden JA, Taylor WF (1986). Papillary thyroid cancer treated at the Mayo Clinic, 1946 through 1970: initial manifestations, pathologic findings, therapy, and outcome. Mayo Clin Proc.

[CR28] McWilliams RR, Giannini C, Hay ID, Atkinson JL, Stafford SL, Buckner JC (2003). Management of brain metastases from thyroid carcinoma: a study of 16 pathologically confirmed cases over 25 years. Cancer.

[CR29] Misaki T, Iwata M, Kasagi K, Konishi J (2000). Brain metastasis from differentiated thyroid cancer in patients treated with radioiodine for bone and lung lesions. Ann Nucl Med.

[CR30] Muacevic A, Wowra B, Siefert A, Tonn JC, Steiger HJ, Kreth FW (2008). Microsurgery plus whole brain irradiation versus Gamma Knife surgery alone for treatment of single metastases to the brain: a randomized controlled multicentre phase III trial. J Neurooncol.

[CR31] Pacak K, Sweeney DC, Wartofsky L, Mark AS, Punja U, Azzam CJ, Burman KD (1998). Solitary cerebellar metastasis from papillary thyroid carcinoma: a case report. Thyroid.

[CR32] Parker LN, Wu SY, Kim DD, Kollin J, Prasasvinichai S (1986). Recurrence of papillary thyroid carcinoma presenting as a focal neurologic deficit. Arch Intern Med.

[CR33] Pazaitou-Panayiotou K, Kaprara A, Chrisoulidou A, Boudina M, Georgiou E, Patakiouta F, Drimonitis A, Vainas I (2005). Cerebellar metastasis as first metastasis from papillary thyroid carcinoma. Endocr J.

[CR34] Richard S, Campello C, Taillandier L, Parker F, Resche F (1998). Haemangioblastoma of the central nervous system in von Hippel–Lindau disease. French VHL Study Group. J Intern Med.

[CR35] Salvati M, Frati A, Rocchi G, Masciangelo R, Antonaci A, Gagliardi FM, Delfini R (2001). Single brain metastasis from thyroid cancer: report of twelve cases and review of the literature. J Neurooncol.

[CR36] Schlumberger MJ (1998). Papillary and follicular thyroid carcinoma. N Engl J Med.

[CR37] Shaha AR, Shah JP, Loree TR (1997). Differentiated thyroid cancer presenting initially with distant metastasis. Am J Surg.

[CR38] Tanaka T, Kato N, Aoki K, Nakamura A, Watanabe M, Arai T, Hasegawa Y, Aoki K, Yamamoto K, Abe T (2013). Cerebellar hemorrhage secondary to cerebellopontine angle metastasis from thyroid papillary carcinoma. Neurol Med Chir (Tokyo).

[CR39] Tsuda K, Tsurushima H, Takano S, Tsuboi K, Matsumura A (2013). Brain metastasis from papillary thyroid carcinomas. Mol Clin Oncol.

[CR40] Tuffaha MSA (2008). Phenotypic and genotypic diagnosis of malignancies: an immunohistochemical and molecular approach.

[CR41] Tunio MA, Al Asiri M, Al-Qahtani KH, AlShakweer W (2015). Cerebellum as initial site of distant metastasis from papillary carcinoma of thyroid: review of three cases. Case Rep Neurol Med.

[CR42] Umehara T, Okita Y, Nonaka M, Mori K, Kanemura Y, Kodama Y, Mano M, Kudawara I, Nakajima S (2015). Choroid plexus metastasis of follicular thyroid carcinoma diagnosed due to intraventricular hemorrhage. Intern Med.

[CR43] Vrachimis A, Schmid KW, Jurgens H, Schober O, Weckesser M, Riemann B (2013). Cerebral metastases from thyroid carcinoma: complete remission following radioiodine treatment. Dtsch Arztebl Int.

